# Broadband X-ray ptychography using multi-wavelength algorithm

**DOI:** 10.1107/S1600577520014708

**Published:** 2021-01-01

**Authors:** Yudong Yao, Yi Jiang, Jeffrey Klug, Youssef Nashed, Christian Roehrig, Curt Preissner, Fabricio Marin, Michael Wojcik, Oliver Cossairt, Zhonghou Cai, Stefan Vogt, Barry Lai, Junjing Deng

**Affiliations:** a Advanced Photon Source, Argonne National Laboratory, IL 60439, USA; b Mathematics and Computer Science Division, Argonne National Laboratory, IL 60439, USA; c Department of Electrical Engineering and Computer Science, Northwestern University, IL 60208, USA

**Keywords:** coherent diffraction imaging, ptychography, partial coherence, high-throughput

## Abstract

Ptychography is a rapidly developing scanning microscopy which is able to view the internal structures of samples at a high resolution beyond the illumination size. The achieved spatial resolution is theoretically dose-limited. A broadband source can provide much higher flux compared with a monochromatic source; however, it conflicts with the necessary coherence requirements of this coherent diffraction imaging technique. In this paper, a multi-wavelength reconstruction algorithm has been developed to deal with the broad bandwidth in ptychography. Compared with the latest development of mixed-state reconstruction approach, this multi-wavelength approach is more accurate in the physical model, and also considers the spot size variation as a function of energy due to the chromatic focusing optics. Therefore, this method has been proved in both simulation and experiment to significantly improve the reconstruction when the source bandwidth, illumination size and scan step size increase. It is worth mentioning that the accurate and detailed information of the energy spectrum for the incident beam is not required in advance for the proposed method. Further, we combine multi-wavelength and mixed-state approaches to jointly solve temporal and spatial partial coherence in ptychography so that it can handle various disadvantageous experimental effects. The significant relaxation in coherence requirements by our approaches allows the use of high-flux broadband X-ray sources for high-efficient and high-resolution ptychographic imaging.

## Abstract 2

Broadband X-ray ptychography is developed based on the multi-wavelength reconstruction method and is systematically demonstrated in both simulation and experiment. In addition, a combined reconstruction approach is proposed to further increase the ptychographic imaging rate.

## 1. Introduction   

Ptychography is a mixture of coherent diffraction imaging (CDI) and scanning microscopy (Rodenburg *et al.*, 2007 ▸; Thibault *et al.*, 2008 ▸; Pfeiffer, 2018 ▸). The achieved spatial resolution of CDI is determined by the maximum scattered angle of photons from samples, which can be much higher than the numerical aperture of the illumination optics. Using a scanning technique with partially overlapping scan spots (Bunk *et al.*, 2008 ▸), CDI is then extended to ptychography to image extended samples. X-ray ptychography has been used to obtain high-resolution quantitative insights of samples in biology (Giewekemeyer *et al.*, 2010 ▸; Deng *et al.*, 2015*a* ▸, 2018 ▸; Diaz *et al.*, 2015 ▸), materials science (Holler *et al.*, 2014 ▸; Hruszkewycz *et al.*, 2012 ▸; Shapiro *et al.*, 2014 ▸; Donnelly *et al.*, 2017 ▸) and electronics (Guizar-Sicairos *et al.*, 2014 ▸; Deng *et al.*, 2017 ▸; Holler *et al.*, 2017 ▸). The redundant information provided by overlapping scan spots also enables the reconstruction of illumination function for X-ray optics characterization (Kewish *et al.*, 2010 ▸; Schropp *et al.*, 2010 ▸) and the correction of scanning position errors (Maiden *et al.*, 2012 ▸; Zhang *et al.*, 2013 ▸; Odstrčil *et al.*, 2018 ▸).

As a coherent imaging technique, ptychography requires an illumination with high spatial and temporal coherence so that the probe can be described as a pure function in the conventional formulation of ptychography reconstruction. However, the available flux would greatly reduce when the illumination source is spatially and spectrally filtered in order to obtain high coherence degree, which in turn affects the quality of ptychography as its resolution is dose-limited (Schropp & Schroer, 2010 ▸; Thibault *et al.*, 2014 ▸). To access higher flux on existing X-ray sources, partially spatial coherent illumination has been demonstrated in CDI (Whitehead *et al.*, 2009 ▸; Clark & Peele, 2011 ▸; Chen *et al.*, 2012 ▸) using the Gaussian–Shell model (Starikov & Wolf, 1982 ▸) in the phase retrieval. A significant relaxation on spatial coherence requirement for ptychography has been recently achieved through a mixed-state (MS) decomposition approach (Thibault & Menzel, 2013 ▸). The decomposed multiple modes in this approach can model the transverse partial coherence in the source as well as other decoherence effects in the sample plane [such as vibration (Clark *et al.*, 2014 ▸) and fly scan (Pelz *et al.*, 2014 ▸; Deng *et al.*, 2015*b* ▸; Huang *et al.*, 2015 ▸)] and the detector plane as well.

Increasing the bandwidth of the illumination is an alternative way to obtain ten times higher flux or more. To reliably phase the whole diffraction pattern, it requires the maximum path length difference (PLD) between waves from the opposite edges of the illumination spot to be smaller than the longitudinal coherence length (Van der Veen & Pfeiffer, 2004 ▸; Enders *et al.*, 2014 ▸; Jacobsen *et al.*, 2017 ▸). Therefore, it imposes a limit on the spectral bandwidth,
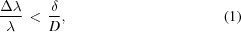
where δ is the spatial resolution corresponding to the maximum solid angle that still carries signals, and *D* is the lateral extension of the illumination on the sample. The aforementioned mixed-state approach has been introduced to reliably reconstruct objects with a broad bandwidth X-ray beam (Enders *et al.*, 2014 ▸). However, the bandwidth together with the beam size used in this study still satisfied equation (1), indicating that the data was mostly free from spectral blurring. As the bandwidth and/or illumination size increase to the extent that equation (1) is no longer valid, reconstruction methods based on monochromatic models including the mixed-state approach may fail, and high-resolution imaging will become more challenging.

To address the effect of broadband illumination in diffractive imaging, a reconstruction method employing multiple wavelengths was proposed in a conventional CDI experiment where a tabletop high-harmonic-generation (HHG) source was used (Chen *et al.*, 2009 ▸). This multi-wavelength (MW) approach assumes that the diffraction pattern with a broadband source is the superposition of diffracted intensities from a series of wavelengths within the spectrum, therefore it is more accurate to represent the physic model of the broadband illumination compared with the mixed-state approach. This polyCDI method was also successfully demonstrated by a synchrotron X-ray source with a spherical-grating monochromator (Abbey *et al.*, 2011 ▸), showing a factor of 60 reduction in the exposure time over monochromatic CDI. X-ray ptychography with broadband illumination has been very attractive to improve the imaging throughput. The extension of this multi-wavelength approach for X-ray ptychography has recently been reported in simulation (Pradier *et al.*, 2016 ▸).

In this paper, we develop broadband X-ray ptychography based on this multi-wavelength approach and systematically demonstrate it in both simulation and experiment. Both simulated and experimental results show that the developed multi-wavelength method has better tolerance of source bandwidth, illumination size and scan step size, producing better reconstruction quality compared with conventional single-mode and latest developed mixed-state methods. Unlike the existing polychromatic reconstruction methods (Chen *et al.*, 2009 ▸; Abbey *et al.*, 2011 ▸; Pradier *et al.*, 2016 ▸) which assume a known spectrum of the incident illumination, our approach does not have such a requirement. Only rough spectral information of the illumination probe, such as the central wavelength and bandwidth, is needed to initialize the spectral probe modes by considering that all probe functions at discretized wavelengths propagate from the same focusing optics and generate wavelength-dependent illumination functions on the sample. The spectral probe modes are updated during the iterative reconstruction, consequently providing the spectral weight for each spectral mode by calculating its power percentage. As the mixed-state approach is able to deal well with various spatial decoherence effects (Thibault & Menzel, 2013 ▸), we further develop a combined approach, in which the mixed-state method is integrated into our developed multi-wavelength reconstruction algorithm. The proposed combined approach can jointly solve partial spatial and temporal coherence in real experiments, such as fly scan using a broadband source, thus significantly increasing the imaging quality and throughput.

## 2. Method   

In ptychography, the object *O*(*r*) is scanned by the illumination beam (which is usually referred to as the probe) *P*(*r*). At the *j*th scan position *r*_*j*_ (*j* = 1, 2, 3,…, *J*, where *J* is the number of total scan points), the complex-valued exit wave from the object is φ(*r*, *r*_*j*_) = *O*(*r*, *r*_*j*_)*P*(*r*). Assuming a monochromatic coherent illumination, the expected diffraction intensities collected by the detector situated in the far field can be expressed as
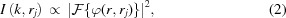
where *k* is the reciprocal coordinate with respect to the real space coordinate *r* in the specimen plane. 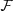 represents the Fourier transform operator. Fully coherent illumination can produce high-contrast speckles in the diffraction pattern, which can be uniquely inverted to determine the structure of the object as long as the feature of the speckles are sampled with a sufficient high frequency.

The diffraction pattern taken under broadband illumination is different because of the imperfect temporal coherent property resulting from the broad bandwidth. As the speckle pattern is wavelength dependent, the acquired diffraction pattern with a broadband illumination is the average of patterns resulting from slightly different wavefronts at different wavelengths, resulting in speckle blurring. Therefore, the development of reconstruction algorithms is needed for broadband ptychography.

### 2.1. Multi-wavelength approach   

The polychromatic diffraction from a broadband illumination is given by the diffraction intensity contributed from each wavelength (Abbey *et al.*, 2011 ▸),
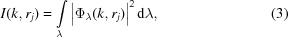
where *I*(*k*, *r*_*j*_) is the diffraction intensity of the *j*th scan position and Φ_λ_ is the diffracted wave at wavelength λ. After discretizing the bandwidth in a small step, equation (3) can be re-written as
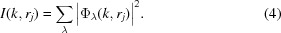
As the incident X-ray energy is away from the elemental absorption edge of the sample, the object transmission function can be assumed to be constant under this broadband illumination, while the probe can be decomposed into a set of incoherent spectral probe modes *P*_λ_(*r*) that are corresponding to a series of wavelengths within the illumination bandwidth,
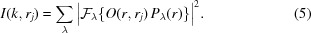
These spectral probe modes are spatially created by the propagation from the focusing optics. In this study, we use a Fresnel zone plate (FZP) which is a chromatic optics (Wang *et al.*, 2003 ▸) that produces variant focal lengths for different wavelengths,
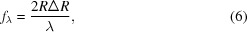
where 2*R* is the diameter of the zone plate and Δ*R* is the outermost zone width. Therefore, the probe profile and size on the sample plane are wavelength-dependent. At the beginning of the reconstruction process, the probe modes are initialized with the numerical propagation from the FZP with the wavelength-dependent focusing property expressed as equation (6). The diffraction propagation from the sample plane to the detector plane is also wavelength-dependent. For this, we need to carefully consider pixel scaling resulting from the wavelength-dependent far-field propagator (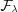) which is implemented via a discrete Fourier transform,
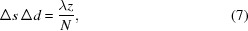
where Δ*s* and Δ*d* are pixel sizes on sample plane and detector plane, respectively, *z* is the distance between these two planes, and *N* is the pixel number used for numerical propagation. In the real space, the array pixel size for a specific λ′ is scaled by λ′/λ_0_ (where λ_0_ is the central wavelength of the spectrum). To keep the real-space pixel size the same for all discretized wavelengths, the rescaling processing is needed, and is achieved by changing the pixel number *N* for different wavelengths in the Fourier transform.

### 2.2. Combined approach of multi-wavelengths and mixed states   

In the real experiments, there are various disadvantageous experimental effects reducing the coherence degree, such as illumination with partially spatial coherence, beam and/or sample fluctuation and drift (Thibault & Menzel, 2013 ▸; Clark *et al.*, 2014 ▸), and detector point spread function. It is worth mentioning that the fly scan technique has been recently implemented in ptychography to reduce the stage overhead between scan points (Pelz *et al.*, 2014 ▸; Deng *et al.*, 2015*b* ▸; Huang *et al.*, 2015 ▸); however, it brings a similar signature of decoherence in far-field diffraction. To deal with those spatial decoherence effects with a broadband illumination, extra orthogonal states *P*_λ,*n*_ are introduced into each chromatic probe mode in the phase-retrieval procedure. Therefore, the far-field diffraction can be described as the superposition of the diffraction intensity from those orthogonal modes at different wavelengths,
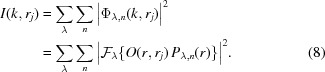
In the reconstruction, the calculated wavefront on the detector plane is updated by a modulus constraint with the measured intensity *I*_M_(*k*, *r*_*j*_),
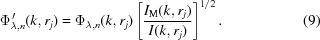
The updated wavefront 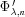 is back-propagated from the detector plane to the object plane, giving the updated exit wave 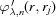. Then the real space updates for both object and probe are applied simultaneously using the following update functions, 
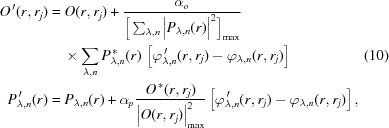
where α_*o*_ and α_*p*_ are the update step sizes and * denotes the complex conjugate. The weighting factor ε_λ_ for wavelength λ can be created from the intensity percentage of each spectral probe mode, 
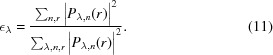



## 3. Numerical simulations   

**Figure 1:**
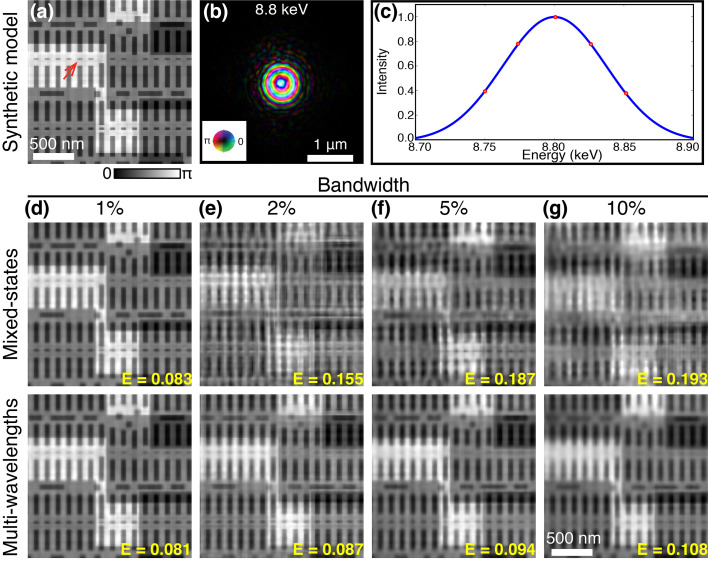
Comparison of two reconstruction strategies with different illumination bandwidths. (*a*) Ground truth phase image of a synthetic sample generated from a chip design file; (*b*) an example probe at the central wavelength (8.8 keV) in the simulation; (*c*) source spectrum with a Gaussian distribution of 1% bandwidth, the red dots indicate the five wavelengthes used in multi-wavelength reconstruction; (*d*)–(*g*) reconstructed phase images for bandwidth of 1%, 2%, 5%, 10%, respectively; images on the top show the results using the mixed-state approach while those at the bottom show the corresponding results using the multi-wavelength method. The normalized reconstruction error *E* is shown on each reconstructed image.

To validate the multi-wavelength method, we first performed the numerical simulations of broadband X-ray ptychography with source energy centered at 8.8 keV. A synthetic sample as shown in Fig. 1 ▸(*a*) was generated from the design file of an integrated circuit with a total thickness of 4.4 µm. The simulated chip contains multiple layered structures made from copper, aluminium, tungsten and silicon, in which the smallest feature (marked by a red arrow) has 10 nm feature size in the vertical direction. All simulations were performed by modeling the spectrum of the broadband illumination as a discrete set of frequency signals with a Gaussian distribution. In order for the illumination function to be closer to the experiment case, a previous reconstructed probe [see Fig. 1 ▸(*b*)] produced by a FZP with 180 µm diameter and 50 nm outermost zone width was used as the probe at the center energy (8.8 keV). The probe functions at different wavelengths were calculated by numerical propagation from this FZP when the sample was placed at a specific position. Variable beam sizes can be obtained by changing the defocus distance. The simulated diffraction patterns were obtained via equation (5) with 101 discretized wavelengths uniformly sampling the spectrum. At each scan position, 5 × 10^6^ photons were incident on the sample assuming an X-ray flux of 5 × 10^8^ photons s^−1^ and an exposure time of 10 ms. The Poisson noise was added to the diffraction pattern. Simulations based on different bandwidths, beam sizes and step sizes were performed.

The simulated datasets were analyzed with the mixed-state and multi-wavelength methods. For the mixed-state method, the probe initialization was performed by creating several shifted copies of the single probe mode (generated by the numerical propagation from the FZP), orthogonalizing them, and taking only the five most powerful modes. For the multi-wavelength method, as introduced in Section 2.1, the spectral probe modes were initialized by performing the wavelength-dependent propagation from the focusing optics to the sample plane. All the probe modes were updated simultaneously with the object using the ePIE algorithm with 100 iterations. Since the ground truth object, denoted *O*(*r*), is known in the simulated case, the image quality of the reconstructed object *O*_*R*_(*r*) can be evaluated using the normalized error (Maiden & Rodenburg, 2009 ▸),
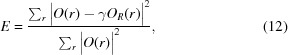
with
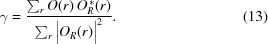



### 3.1. Bandwidth   

Firstly, ptychography with different illumination bandwidths were simulated to investigate the effect of the bandwidth. The sample was placed about 300 µm downstream of the focus position of 8.8 keV X-rays. The probe profile at the central wavelength is shown in Fig. 1 ▸(*b*). The sample was raster-scanned with 100 nm step size, covering a 2 µm × 2 µm field-of-view. 1%, 2%, 5% and 10% bandwidth were used to generate the diffraction patterns in ptychography scans. Both mixed-state and multi-wavelength approaches were used in reconstructions to compare the tolerance of the bandwidth. For 1% bandwidth, the mixed-state method was performed using five orthogonal probe modes, while the multi-wavelength method used five wavelengths centering at 8.8 keV with 25 eV interval, as marked in Fig. 1 ▸(*c*). The reconstructed phase images using 1% bandwidth illumination are shown in Fig. 1 ▸(*d*). Both approaches give similar reconstruction quality that is very close to the ground truth image [Fig. 1 ▸(*a*)]. As the bandwidth of the illumination increases, 5, 10 and 15 orthogonal probe modes were used in the mixed-state method to process the 2%, 5% and 10% bandwidth datasets, and the same numbers of spectral modes were implemented when using the multi-wavelength approach. The normalized reconstruction error of the retrieved object was calculated by equation (12) and displayed on each reconstructed image. As shown in Figs. 1 ▸(*e*)–1(*g*), the mixed-state method fails to obtain a high-quality reconstruction as the bandwidth increases. In comparison, the multi-wavelength approach yields better reconstruction, showing that this approach has better tolerance of the bandwidth.

### 3.2. Beam size   

**Figure 2:**
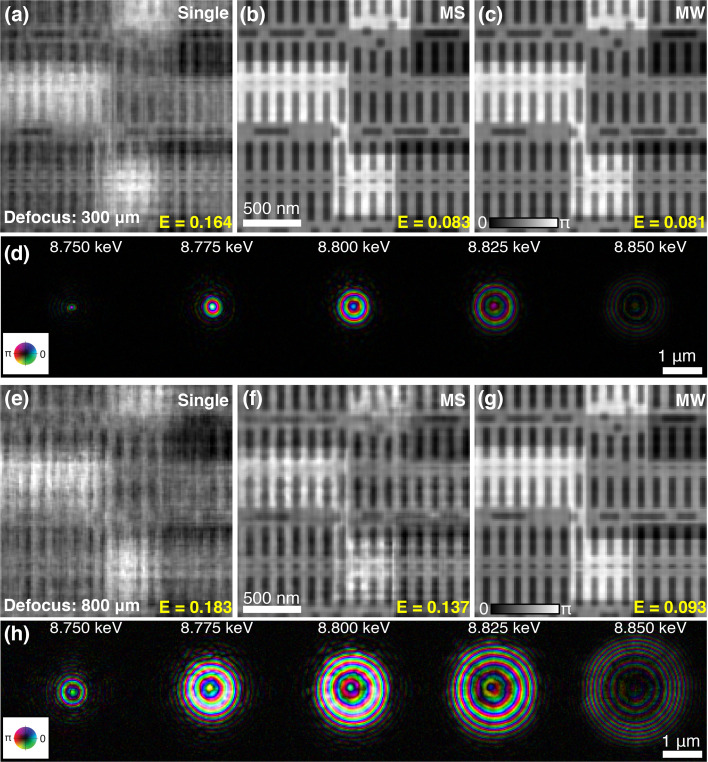
Reconstruction comparison with different beam sizes. A ptychographic dataset acquired with 500 nm beam size is reconstructed by single-mode ptychography (*a*), mixed-state (MS) method (*b*) and multi-wavelength (MW) method (*c*). Panel (*d*) shows the simultaneously reconstructed five spectral probe modes during the reconstruction of (*c*). Panels (*e*)–(*g*) are reconstructions for 1.5 µm illumination beam size using single-mode ptychography, MS method and MW method, respectively. Panel (*h*) shows the five spectral probe modes obtained simultaneously in the reconstruction of (*g*).

As indicated in equation (1), the probe size also affects ptychography with a broadband source. By placing the sample at two longitudinal positions (300 µm and 800 µm downstream of the focal spot of 8.8 keV X-rays), 500 nm and 1.5 µm beam sizes were obtained with 1% bandwidth illumination. The beam size is determined by the full width at half-maximum (FWHM) of the superposition of the probes at different wavelengths. For these two cases, the sample was scanned with 100 nm and 300 nm step size, respectively, to keep a similar linear overlapping ratio (Bunk *et al.*, 2008 ▸). Three reconstruction methods (the conventional single-mode approach, mixed-state approach and multi-wavelength approach) were performed. Figs. 2 ▸(*a*)–2(*c*) show the corresponding reconstructions of the dataset acquired with 500 nm probe size. As expected, conventional ptychography using the single mode is unable to reconstruct a faithful image [Fig. 2 ▸(*a*)]. The introduction of five orthogonal modes in the mixed-state method greatly improves the reconstruction quality, with smallest features (∼10 nm) clearly resolved [Fig. 2 ▸(*b*)]. Multi-wavelength reconstruction with five wavelengths also gives high-quality results [Fig. 2 ▸(*c*)]. Fig. 2 ▸(*d*) shows the reconstructed probes of these five wavelengths, from which we can find that the probe size is wavelength-dependent, and the intensity of the probe is consistent with the spectrum shown in Fig. 1 ▸(*c*). As the probe size increases to 1.5 µm, equation (1) is no longer satisfied in order to achieve 10 nm spatial resolution. Therefore, the reconstruction quality of both single-mode [Fig. 2 ▸(*e*)] and multi-mode [Fig. 2 ▸(*f*)] methods obviously degrades. Compared with these two reconstruction approaches, the multi-wavelength method notably improves the image quality as shown in Fig. 2 ▸(*g*), with the reconstructed probes at five wavelengths shown in Fig. 2 ▸(*h*). This result indicates that the multi-wavelength approach can better deal with the speckle blurring effect when a larger beam size is used in broadband ptychography.

However, minor quality degradation can be found in Fig. 2 ▸(*g*) when compared with the small beam’s result [Fig. 2 ▸(*c*)] which was reconstructed by the same multi-wavelength approach. This can be attributed to the decrease of the oversampling ratio (Spence *et al.*, 2004 ▸) for large beams as shown in Fig. 2 ▸(*h*). In the simulation, a detector with a pixel size of 75 µm and a sample-to-detector distance of 1.92 m was assumed in order to be close to actual experiments, resulting in about 7.2 and 2.4 sampling ratio in one dimension for 500 nm and 1.5 µm beam, respectively. To improve the oversampling ratio for a large beam, a detector with smaller pixel size or a bigger detector placed further from the sample is suggested. In addition, the large-beam ptychography used a bigger scan step size in order to keep the same linear overlapping ratio as the small-beam ptychography; the reduced dose (about nine times) on the sample also affects the reconstruction quality.

### 3.3. Step size   

**Figure 3:**
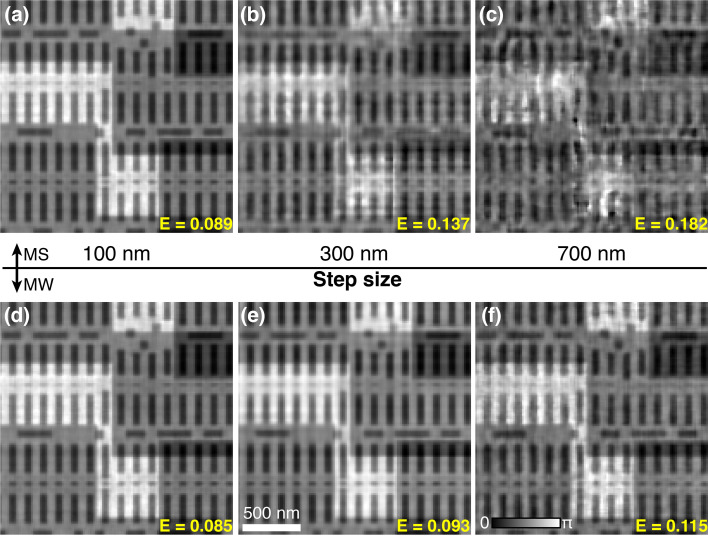
Reconstruction comparison with different step sizes. (*a*)–(*c*) Reconstructed phase images using the mixed-state approach for three ptychography scans with 100 nm (*a*), 300 nm (*b*), 700 nm (*c*) step size. (*d*)–(*f*) Reconstructed phase images using the multi-wavelength approach for the same three datasets with 100 nm (*d*), 300 nm (*e*), 700 nm (*f*) step size.

Scan step size affects the overlapping ratio in ptychography (Bunk *et al.*, 2008 ▸). The increase of overlap through reducing step size generally results in better information redundancy in the recorded diffraction patterns, which helps the convergence of iterative phase retrieval. However, a small step size needs more scan points to cover the same area, which would slow down the data acquisition especially when the stage overhead plays an important role in the step scan ptychography. In addition, more data points requires more computation resource. All of these conflict the goal of high-throughput ptychography. In this section, broadband ptychography with different step sizes were simulated. The bandwidth used is 1%. The same area on the synthetic sample was scanned using a defocused probe with 1.5 µm diameter as shown in Fig. 2 ▸(*h*). Three ptychography scans were acquired with 100 nm, 300 nm and 700 nm step size, respectively. Figs. 3 ▸(*a*)–3(*c*) show the reconstruction results using the mixed-state approach: the reconstruction quality clearly improves with a small step size (100 nm) and then degrades quickly as the step size increases, indicating that the mixed-state method relies on a high overlapping ratio to deal with the broadband illumination. The reconstructed images using the multi-wavelength method are displayed in Figs. 3 ▸(*d*)–3(*f*) with clear improvement in larger-step-size datasets when compared with Figs. 3(*b*) and 3(*c*) ▸ that used the mixed-state approach. This indicates that our multi-wavelength approach has less requirement on the overlapping ratio to solve the imperfect coherence caused by the broadband illumination, which helps to further improve the ptychographic throughput.

## 4. Experimental results   

**Figure 4:**
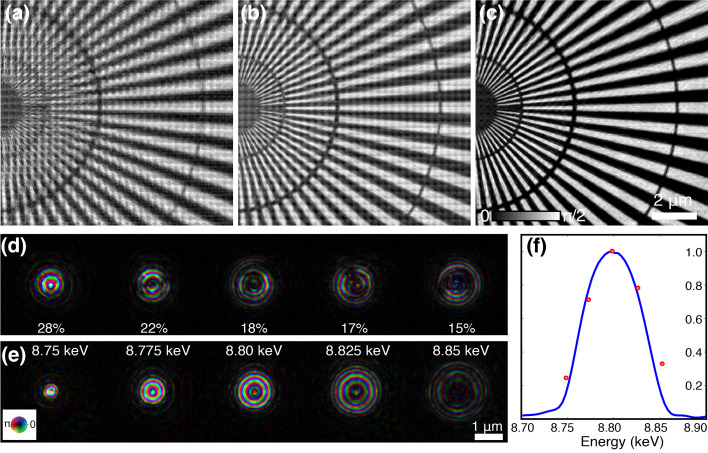
Experimental broadband ptychography using a DMM source with 1% bandwidth. The phase image of a Siemens star sample reconstructed by (*a*) single-mode ptychography reconstruction, (*b*) mixed-state approach, and (*c*) multi-wavelength approach. (*d*) Five orthogonal probe modes reconstructed together with (*b*), the inset values show the power percentage of each mode. (*e*) Five spectral probe modes from the reconstruction of (*c*), with its relative power percentage marked by the red dots in (*f*). The blue curve in (*f*) shows the measured spectrum of this DMM source in the experiment.

To evaluate the effectiveness of the proposed method, a broadband ptychography experiment was performed on the Velociprobe (Deng *et al.*, 2019 ▸) at the Advanced Photon Source (APS), Argonne National Laboratory. A double-multilayer monochromator (DMM) was used to produce X-rays with a spectrum bandwidth of about 1% at the peak energy of 8.8 keV [see Fig. 4 ▸(*f*)]. Then this broadband illumination was focused by a zone plate having the same parameters as that used in the simulation (180 µm diameter and 50 nm outer-most zone width). A gold Siemens star test pattern was placed downstream of the FZP focus plane, resulting in a FWHM beam size of about 1 µm. A step raster scan was conducted with 300 nm step size. The far-field diffraction patterns were acquired with a Dectris Eiger 500K detector (75 µm pixel size) which was placed 1.92 m downstream of the sample. To avoid the counting rate saturation of the detector, an aluminium filter with a thickness of 250 µm was inserted upstream of the zone plate, which cut off about 91% of the X-ray flux, yielding ∼9 × 10^8^ photon s^−1^ on the sample. The exposure time for each diffraction pattern was 10 ms.

Fig. 4 ▸ shows reconstructions of this ptychography scan using three reconstruction approaches. The probe initialization process is the same as that described in simulation, and all the probe modes and object were updated simultaneously at every iteration in the reconstruction with 200 iterations. The phase image reconstructed by conventional single-mode ptychography has lots of artifacts, as shown in Fig. 4 ▸(*a*). The image quality is slightly improved by the mixed-state reconstruction method with five orthogonal probe modes [Fig. 4 ▸(*d*)], but still contains obvious grid artifacts in Fig. 4 ▸(*b*). A reconstruction using ten orthogonal modes was also performed, but no improvement was observed with the increasing number of probe modes. Then the multi-wavelength approach was implemented using five wavelengths 25 eV apart as marked in Fig. 4 ▸(*f*). The reconstructed phase image in Fig. 4 ▸(*c*) has a much improved quality with the central spokes of 30 nm clearly resolved, and free of artifacts. Fig. 4 ▸(*e*) displays the reconstructed probe functions at these five wavelengths, clearly showing that the probe size is wavelength dependent. The size difference between those spectral probe modes is proportional to the bandwidth divided by the number of the probe modes, which is about 360 nm with a 180 µm-diameter zone plate. This wavelength-dependent variation helps the reconstruction algorithm to seperate the longitudinal modes. The power intensity of the five spectral probe modes is normalized to the spectrum [see Fig. 4 ▸(*f*)], and is consistent with the measured bandwidth distribution.

### 4.1. Beam size   

**Figure 5:**
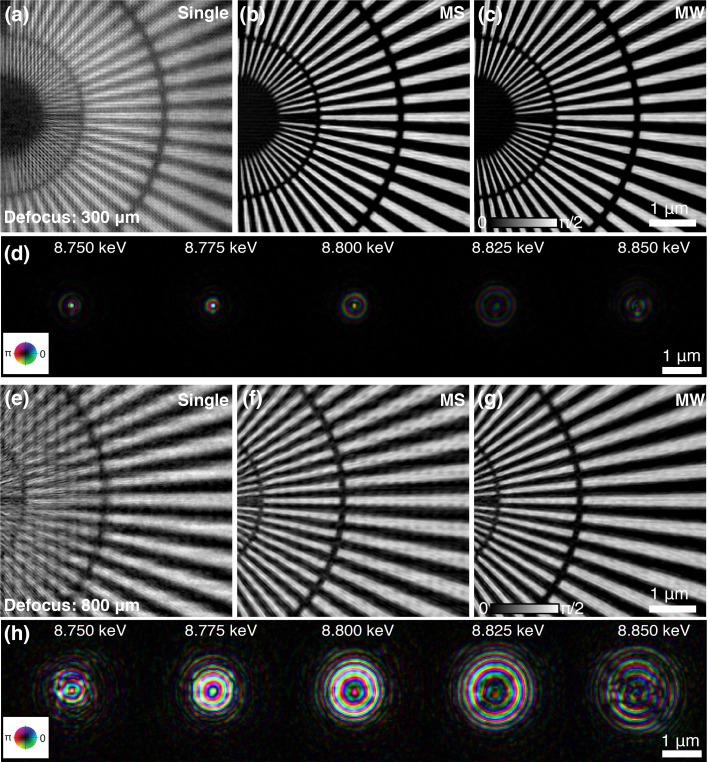
Broadband ptychography using different beam sizes. The probe size was changed by placing the sample at different defocus positions. Panels (*a*)–(*d*) show ptychography reconstructions of a dataset acquired with a ∼500 nm illumination of 1% bandwidth, using single-mode (*a*), mixed-state approach (*b*), multi-wavelength approach (*c*). The reconstructed five spectral probe modes are shown in (*d*). Panels (*e*)–(*h*) are corresponding reconstructions using a 1.5 µm illumination size.

To validate the simulation results of broadband ptychography with different probe sizes, the test sample was placed at two defocus positions (300 µm and 800 µm downstream of the focus position of 8.8 keV X-rays), generating an illumination on the sample with a FWHM size of about 500 nm and 1.5 µm, respectively. For the 500 nm illumination, 100 nm step size was used for the ptychography scan, while 300 nm step size was used for 1.5 µm illumination to keep the same oversampling ratio. The exposure time of both scans was 10 ms. Figs. 5 ▸(*a*) and 5(*e*) show the reconstruction using the conventional single-mode ptychographic algorithm, which assumes full coherence of the illumination beam. The quality of the reconstruction is clearly degraded due to the partial coherence effect caused by the broadband illumination. As shown in the images, there is a transverse shift between the scan area in these two datasets – this is because the translation stage to place the sample at defocus positions is not exactly parallel to the X-ray beam direction. The reconstructions using the mixed-state method on these two scans are shown in Figs. 5 ▸(*b*) and 5(*f*). Compared with Figs. 5 ▸(*a*) and 5(*e*), the image quality is notably improved. However, the mixed-state approach still does not give a converged result when using 1.5 µm beam size, which can be seen in Fig. 5 ▸(*f*) with some evident artifacts. As explained in the simulation, the requirement imposed by equation (1) becomes more challenging as the probe size increases, and the mixed-state approach based on spatial decomposition starts to fail. Figs. 5 ▸(*c*) and 5(*g*) are reconstructions using the multi-wavelength approach. For the small illumination of 500 nm, the multi-wavelength approach gives similar reconstruction quality as the mixed-state approach, while it gives much higher reconstruction quality for the 1.5 µm illumination case when compared with the mixed-state reconstruction. This shows that the multi-wavelength approach based on spectral decomposition can better handle the broadband illumination with a large beam size.

### 4.2. Step size   

**Figure 6:**
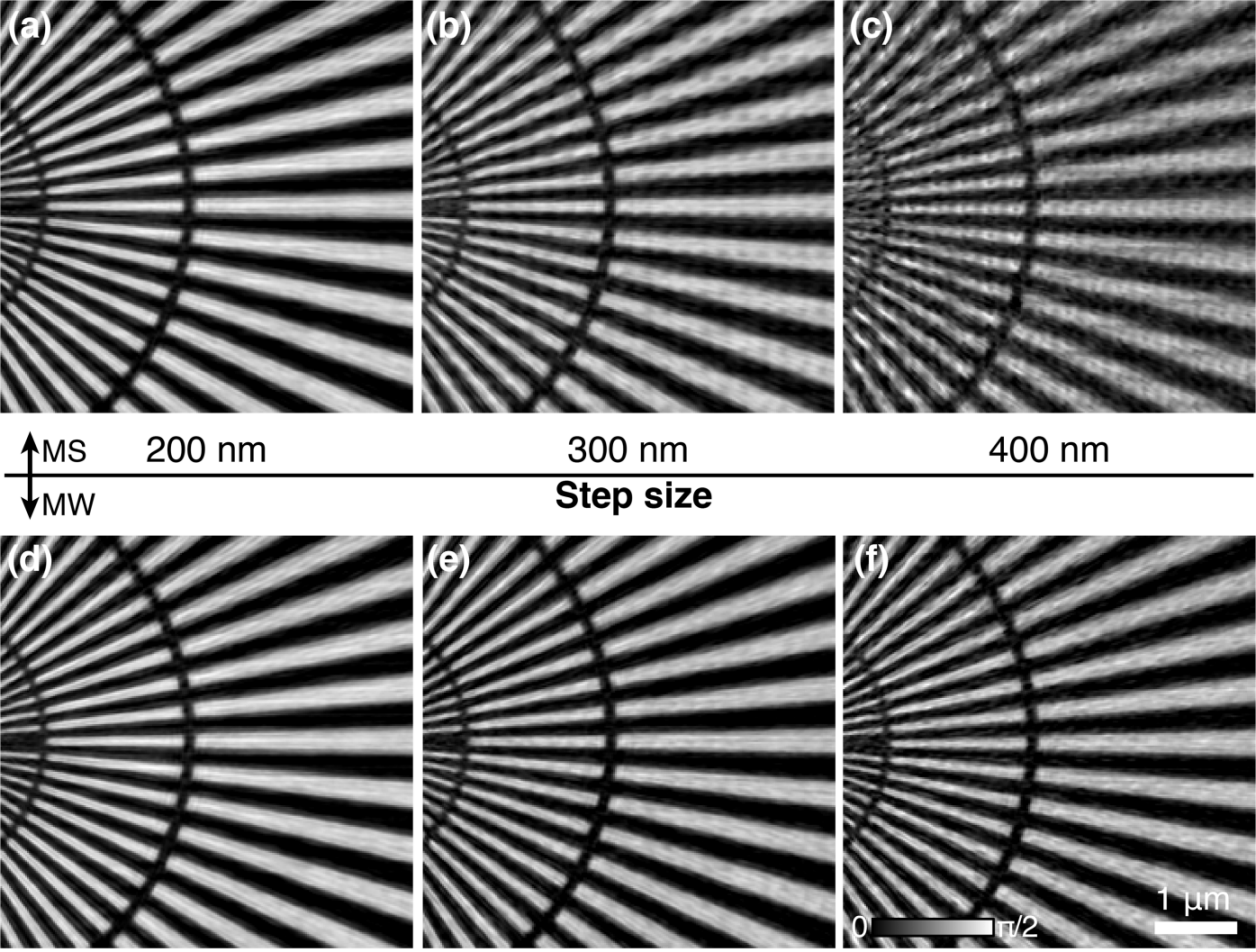
Broadband ptychography with different step size. Three ptychography scans with 1.5 µm illumination were conducted using 200 nm, 300 nm, 400 nm step size. Panels (*a*)–(*c*) show reconstructions using the mixed-state approach. Panels (*d*)–(*f*) are the corresponding reconstructions using the multi-wavelength approach.

The simulation shows that the proposed multi-wavelength method helps increase the allowable scan step size compared with the mixed-state method when using a broadband illumination. The verification experiment was carried out with three ptychography scans using 200 nm, 300 nm, 400 nm step size and 1.5 µm beam size. Figs. 6 ▸(*a*)–6(*c*) are the reconstruction results using the mixed-state method with five probe modes, showing that the image quality degrades as the step size increases. The corresponding reconstructions by the multi-wavelength method in Figs. 6 ▸(*d*)–6(*f*) show that the multi-wavelength approach has better performance in broadband ptychography as the scan step size increases.

### 4.3. Combine approach for broadband fly scan ptychography   

**Figure 7:**
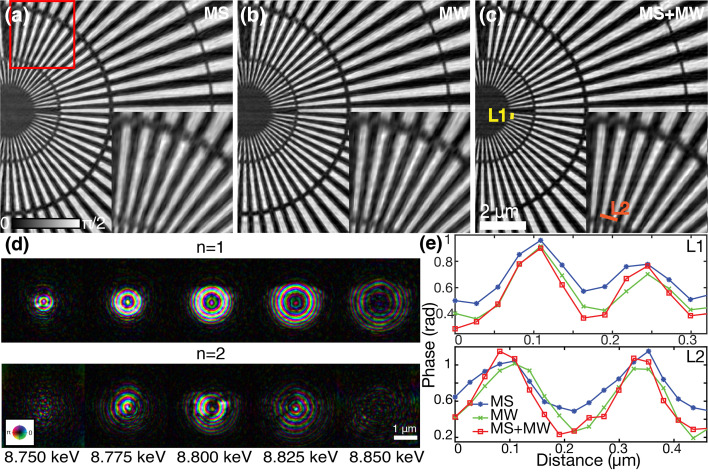
Broadband ptychography implemented in fly scan. (*a*) Mixed-state reconstruction using five orthogonal probe modes; (*b*) multi-wavelength reconstruction using five wavelengths; (*c*) combined approach with five wavelengths and two orthogonal modes at each wavelength [insets show a zoomed region denoted by the red box in (*a*)]; (*d*) reconstructed probes by the combined method, the five columns are corresponding to five wavelengths in the reconstruction, each column contains two orthogonal probe modes (*n* = 1, 2) at each wavelength; (*e*) line profiles for the selected positions marked by the yellow (L1) and orange (L2) lines in (*c*), respectively.

In the practical experiment, there are various imperfect conditions affecting the spatial coherence. The mixed-state approach is a good tool to handle these decoherence effects. For example, this approach has been used in fly scan ptychography with a monochromatic beam to deal with the speckle blurring due to the continuous motion of the sample in the scan (Deng *et al.*, 2015*b* ▸). To demonstrate the proposed combination approach of the multi-wavelength and mixed-state method, a fly scan ptychography using 1% bandwidth was conducted in a snake-scan trajectory (Deng *et al.*, 2019 ▸) with a step size of 100 nm and 600 nm in the horizontal (fast) and vertical (slow) axis, respectively. The Eiger 500K detector was triggered with a frequency of 100 frames s^−1^. Three reconstruction strategies were used to process the data: the mixed-state method with five orthogonal probe modes [Fig. 7 ▸(*a*)], the multi-wavelength method with five wavelengths [Fig. 7 ▸(*b*)], and the combined method with five wavelengths and two orthogonal modes per wavelength [Fig. 7 ▸(*c*)]. Compared with Figs. 7 ▸(*a*) and 7(*b*), the phase image in Fig. 7 ▸(*c*) reconstructed by the proposed combined approach gives better quality with sharper features, revealing that this combined approach is able to simultaneously solve the imperfect partial coherence caused by both fly scan and broadband illumination. The reconstructed probes using the combined method are shown in Fig. 7 ▸(*d*). One can observe the increase of the beam size for the five sampled energies from left to right, which is consistent with the focusing behavior by chromatic optics and above reconstruction results. At each column, the top shows the first orthogonal mode (*n* = 1) which contains most of the power intensity while the bottom shows the second orthogonal mode (*n* = 2). As the wavelength is away from the central wavelength, the contribution of the second probe mode becomes less. The line-cut profiles for Figs. 7 ▸(*a*)–7(*c*) from the selected positions marked in Fig. 7 ▸(*c*) are given in Fig. 7 ▸(*e*), showing that the combined method provided better image contrast and sharper edge compared with the mixed-state method and the multi-wavelength method.

## 5. Conclusion   

A multi-wavelength approach was incorporated into the reconstruction of broadband ptychography by decomposing the probe into a set of chromatic probe modes which are generated using the numerical propagation from the focusing optics. The object and probe functions at multiple wavelengths can be reconstructed simultaneously during the iterative algorithm. An accurately known spectrum of the broadband illumination is not necessary for this developed reconstruction method while more accurate initial input of the weighting factor of each wavelength can help the reconstruction convergence. Although currently the multi-wavelength method can only recover the weighting factor of a few discretized wavelengths in the spectrum, its accuracy and the energy resolution may be both improved for the recovery of the full illumination spectrum by further algorithm development, for example implementing the constraint during the probe update that all spectral probe modes have the same distribution before the focusing optics.

We have systematically shown in both simulation and experiments that our multi-wavelength method can improve the tolerance of bandwidth, beam size and step size, compared with the conventional single-mode ptychography method and the mixed-state method. One of the main advantages of the proposed method is that it can take more incident flux from a broadband illumination to speed up data acquisition speed. The DMM source used in the experimental demonstration can provide more than 20 times higher flux compared with a double-crystal monochromator (DCM) source in our beamline. However, only a factor of about five reduction in the exposure time was achieved on the low-absorption test sample due to the use of a filter to protect the detector. The filter can be moved away if the detector does not saturate with a thicker sample. For example, a recent study on a thick integrated circuit without the filter was demonstrated to make full use of the high flux of this DMM source (Deng *et al.*, 2019 ▸). For more general samples, a semi-transparent central stop in front of the detector is currently being tested to attenuate the primary beam and thus to solve the counting-rate saturation issue. In future, the development of a photon-counting detector with a higher count rate or photon-integrating detector with high performance will also help to make full use of the high flux provided by broadband sources.

In this study, we also further integrated the mixed-state method into the multi-wavelength reconstruction procedure to jointly solve the partial temporal and spatial coherent problem simultaneously. A fly scan ptychography experiment was performed to demonstrate this combined model. With this combined approach, the throughput of high spatial resolution ptychographic imaging can be increased by one to two orders of magnitude by combining, for example, fly scan with broadband illumination, or relaxing the requirements of spectral and spatial filtering for coherent X-ray sources. The upcoming APS upgrade will provide at least 100 times coherent flux with the source spot size and emittance in the horizontal direction squeezed down to values similar to those of the vertical. With the improvement of horizontal coherence degree, the proposed combined approach is potentially able to directly use the broadband source from the undulator without any spectral and spatial filtering between the source and the endstation. Therefore, a combination of a thousand-fold improvement in X-ray flux can be expected when using the broadband illumination approach after the APS upgrade, which will significantly improve the throughput of X-ray ptychography in terms of speed and resolution.

## References

[bb1] Abbey, B., Whitehead, L. W., Quiney, H. M., Vine, D. J., Cadenazzi, G. A., Henderson, C. A., Nugent, K. A., Balaur, E., Putkunz, C. T., Peele, A. G., Williams, G. & McNulty, I. (2011). *Nat. Photon.* **5**, 420–424.

[bb2] Bunk, O., Dierolf, M., Kynde, S., Johnson, I., Marti, O. & Pfeiffer, F. (2008). *Ultramicroscopy*, **108**, 481–487. 10.1016/j.ultramic.2007.08.003 17764845

[bb3] Chen, B., Abbey, B., Dilanian, R., Balaur, E., van Riessen, G., Junker, M., Tran, C. Q., Jones, M. W. M., Peele, A. G., McNulty, I., Vine, D. J., Putkunz, C. T., Quiney, H. M. & Nugent, K. A. (2012). *Phys. Rev. B*, **86**, 235401.

[bb4] Chen, B., Dilanian, R. A., Teichmann, S., Abbey, B., Peele, A. G., Williams, G. J., Hannaford, P., Van Dao, L., Quiney, H. M. & Nugent, K. A. (2009). *Phys. Rev. A*, **79**, 023809.

[bb5] Clark, J., Huang, X., Harder, R. & Robinson, I. (2014). *Phys. Rev. Lett.* **112**, 113901. 10.1103/PhysRevLett.112.113901 24702370

[bb6] Clark, J. N. & Peele, A. G. (2011). *Appl. Phys. Lett.* **99**, 154103.

[bb7] Deng, J., Hong, Y. P., Chen, S., Nashed, Y. S. G., Peterka, T., Levi, A. J. F., Damoulakis, J., Saha, S., Eiles, T. & Jacobsen, C. (2017). *Phys. Rev. B*, **95**, 104111. 10.1103/PhysRevB.95.104111 PMC5525020 28752135

[bb8] Deng, J., Lo, Y. H., Gallagher-Jones, M., Chen, S., Pryor, A., Jin, Q., Hong, Y. P., Nashed, Y. S. G., Vogt, S., Miao, J. & Jacobsen, C. (2018). *Sci. Adv.* **4**, eaau4548. 10.1126/sciadv.aau4548 PMC6214637 30406204

[bb9] Deng, J., Nashed, Y. S. G., Chen, S., Phillips, N. W., Peterka, T., Ross, R., Vogt, S., Jacobsen, C. & Vine, D. J. (2015*b*). *Opt. Express*, **23**, 5438–5451. 10.1364/OE.23.005438 PMC4394751 25836777

[bb10] Deng, J., Preissner, C., Klug, J. A., Mashrafi, S., Roehrig, C., Jiang, Y., Yao, Y., Wojcik, M., Wyman, M., Vine, D., Yue, K., Chen, S., Mooney, T., Wang, M., Feng, Z., Jin, D., Cai, Z., Lai, B. & Vogt, S. (2019). *Rev. Sci. Instrum.* **90**, 083701. 10.1063/1.5103173 31472643

[bb11] Deng, J., Vine, D. J., Chen, S., Nashed, Y. S. G., Jin, Q., Phillips, N. W., Peterka, T., Ross, R., Vogt, S. & Jacobsen, C. J. (2015*a*). *Proc. Natl Acad. Sci. USA*, **112**, 2314–2319. 10.1073/pnas.1413003112 PMC4345580 25675478

[bb12] Diaz, A., Malkova, B., Holler, M., Guizar-Sicairos, M., Lima, E., Panneels, V., Pigino, G., Bittermann, A. G., Wettstein, L., Tomizaki, T., Bunk, O., Schertler, G., Ishikawa, T., Wepf, R. & Menzel, A. (2015). *J. Struct. Biol.* **192**, 461–469. 10.1016/j.jsb.2015.10.008 26470812

[bb13] Donnelly, C., Guizar-Sicairos, M., Scagnoli, V., Gliga, S., Holler, M., Raabe, J. & Heyderman, L. J. (2017). *Nature*, **547**, 328–331. 10.1038/nature23006 28726832

[bb14] Enders, B., Dierolf, M., Cloetens, P., Stockmar, M., Pfeiffer, F. & Thibault, P. (2014). *Appl. Phys. Lett.* **104**, 171104.

[bb15] Giewekemeyer, K., Thibault, P., Kalbfleisch, S., Beerlink, A., Kewish, C. M., Dierolf, M., Pfeiffer, F. & Salditt, T. (2010). *Proc. Natl Acad. Sci. USA*, **107**, 529–534. 10.1073/pnas.0905846107 PMC2795774 20018650

[bb16] Guizar-Sicairos, M., Johnson, I., Diaz, A., Holler, M., Karvinen, P., Stadler, H. C., Dinapoli, R., Bunk, O. & Menzel, A. (2014). *Opt. Express*, **22**, 14859–14870. 10.1364/OE.22.014859 24977581

[bb17] Holler, M., Diaz, A., Guizar-Sicairos, M., Karvinen, P., Färm, E., Härkönen, E., Ritala, M., Menzel, A., Raabe, J. & Bunk, O. (2014). *Sci. Rep.* **4**, 3857. 10.1038/srep03857 PMC3900995 24457289

[bb18] Holler, M., Guizar-Sicairos, M., Tsai, E. H. R., Dinapoli, R., Müller, E., Bunk, O., Raabe, J. & Aeppli, G. (2017). *Nature*, **543**, 402–406. 10.1038/nature21698 28300088

[bb19] Hruszkewycz, S. O., Holt, M. V., Murray, C. E., Bruley, J., Holt, J., Tripathi, A., Shpyrko, O. G., McNulty, I., Highland, M. J. & Fuoss, P. H. (2012). *Nano Lett.* **12**, 5148–5154. 10.1021/nl303201w 22998744

[bb20] Huang, X., Lauer, K., Clark, J. N., Xu, W., Nazaretski, E., Harder, R., Robinson, I. K. & Chu, Y. S. (2015). *Sci. Rep.* **5**, 9074. 10.1038/srep09074 PMC4357920 25766519

[bb21] Jacobsen, C., Deng, J. & Nashed, Y. (2017). *J. Synchrotron Rad.* **24**, 1078–1081. 10.1107/S1600577517009869 PMC5580791 28862631

[bb22] Kewish, C. M., Thibault, P., Dierolf, M., Bunk, O., Menzel, A., Vila-Comamala, J., Jefimovs, K. & Pfeiffer, F. (2010). *Ultramicroscopy*, **110**, 325–329. 10.1016/j.ultramic.2010.01.004 20116927

[bb23] Maiden, A. & Rodenburg, J. (2009). *Ultramicroscopy*, **109**, 1256–1262. 10.1016/j.ultramic.2009.05.012 19541420

[bb24] Maiden, A. M., Humphry, M. J., Sarahan, M. C., Kraus, B. & Rodenburg, J. M. (2012). *Ultramicroscopy*, **120**, 64–72. 10.1016/j.ultramic.2012.06.001 22813888

[bb25] Odstrčil, M., Menzel, A. & Guizar-Sicairos, M. (2018). *Opt. Express*, **26**, 3108–3123. 10.1364/OE.26.003108 29401843

[bb26] Pelz, P. M., Guizar-Sicairos, M., Thibault, P., Johnson, I., Holler, M. & Menzel, A. (2014). *Appl. Phys. Lett.* **105**, 251101.

[bb27] Pfeiffer, F. (2018). *Nat. Photon.* **12**, 9–17.

[bb28] Pradier, S. R. R., van Riessen, G., Cadenazzi, G. A., Balaur, E., Abbey, B. & Quiney, H. M. (2016). *AIP Conf. Proc.* **1696**, 020048.

[bb29] Rodenburg, J., Hurst, A., Cullis, A., Dobson, B., Pfeiffer, F., Bunk, O., David, C., Jefimovs, K. & Johnson, I. (2007). *Phys. Rev. Lett.* **98**, 034801. 10.1103/PhysRevLett.98.034801 17358687

[bb30] Schropp, A., Boye, P., Feldkamp, J. M., Hoppe, R., Patommel, J., Samberg, D., Stephan, S., Giewekemeyer, K., Wilke, R. N., Salditt, T., Gulden, J., Mancuso, A. P., Vartanyants, I. A., Weckert, E., Schöder, S., Burghammer, M. & Schroer, C. G. (2010). *Appl. Phys. Lett.* **96**, 091102.

[bb31] Schropp, A. & Schroer, C. G. (2010). *New J. Phys.* **12**, 035016.

[bb32] Shapiro, D. A., Yu, Y.-S., Tyliszczak, T., Cabana, J., Celestre, R., Chao, W., Kaznatcheev, K., Kilcoyne, A. L. D., Maia, F., Marchesini, S., Meng, Y. S., Warwick, T., Yang, L. L. & Padmore, H. A. (2014). *Nat. Photon.* **8**, 765–769.

[bb33] Spence, J., Weierstall, U. & Howells, M. (2004). *Ultramicroscopy*, **101**, 149–152. 10.1016/j.ultramic.2004.05.005 15450660

[bb34] Starikov, A. & Wolf, E. (1982). *J. Opt. Soc. Am.* **72**, 923–928.

[bb35] Thibault, P., Dierolf, M., Menzel, A., Bunk, O., David, C. & Pfeiffer, F. (2008). *Science*, **321**, 379–382. 10.1126/science.1158573 18635796

[bb36] Thibault, P., Guizar-Sicairos, M. & Menzel, A. (2014). *J. Synchrotron Rad.* **21**, 1011–1018. 10.1107/S1600577514015343 PMC4181642 25177990

[bb37] Thibault, P. & Menzel, A. (2013). *Nature*, **494**, 68–71. 10.1038/nature11806 23389541

[bb38] Veen, F. & Pfeiffer, F. (2004). *J. Phys. Condens. Matter*, **16**, 5003–5030.

[bb39] Wang, Y., Yun, W. & Jacobsen, C. (2003). *Nature*, **424**, 50–53. 10.1038/nature01756 12840754

[bb40] Whitehead, L. W., Williams, G. J., Quiney, H. M., Vine, D. J., Dilanian, R. A., Flewett, S., Nugent, K. A., Peele, A. G., Balaur, E. & McNulty, I. (2009). *Phys. Rev. Lett.* **103**, 243902. 10.1103/PhysRevLett.103.243902 20366201

[bb41] Zhang, F., Peterson, I., Vila-Comamala, J., Diaz, A., Berenguer, F., Bean, R., Chen, B., Menzel, A., Robinson, I. K. & Rodenburg, J. M. (2013). *Opt. Express*, **21**, 13592–13606. 10.1364/OE.21.013592 23736612

